# Bioprospecting perchlorate-reducing bacteria: report of the genera *Psychrobacter, Planococcus, Carnobacterium, *and* Paeniglutamicibacter* isolated in Cierva Point, Antarctica, and its contribution to SDG 3

**DOI:** 10.1007/s10532-026-10400-z

**Published:** 2026-10-24

**Authors:** Dainis Puentes-Martínez, Julieta Florez-Cuadrado, Leidy Martínez-Los nuevos, Valentina Cardona-Martelo, Yacira Cassiani-Arroyo, Isis Hernández-Rocha, Carolina Rubiano-Labrador, Andrea Monroy-Licht, Rosa Acevedo-Barrios

**Affiliations:** 1 Grupo de Estudios Químicos y Biológicos, Dirección de Ciencias Básicas, Universidad Tecnológica de Bolívar, 130001 Cartagena de Indias D. T. y C, Colombia; 2 Chemistry and Biology Group, Chemistry and Biology Department, Universidad del Norte, 081007 Barranquilla, Colombia

**Keywords:** Antarctica, Perchlorate, Psychrotolerant bacteria, Halotolerant bacteria, Bioremediation, Marine sediments, Emerging contaminants

## Abstract

Perchlorate (ClO_4_^−^) is a global contaminant that has been detected in various environmental matrices. ClO_4_^−^ is considered an emerging pollutant because of its potent endocrine-disrupting effects, which impairs iodine uptake in the thyroid gland, and its high toxicity and persistence. Therefore, the development of technologies for its reduction in ecosystems is imperative; bioremediation is a promising technology for ClO_4_^−^ reduction. In this study, bacterial strains isolated from marine sediments at Cierva Point, an Antarctic Specially Protected Area (ASPA No. 134), were characterized. The methodology consisted of four stages: i) isolation of the bacterial strains; ii) characterization of isolated strains by morphological, biochemical, and molecular traits; iii) susceptibility tests against NaCl and ClO_4_^−^; iv) ClO_4_^−^ removal assays using a selective electrode. The strains grew at 10 °C for 7 days and tolerated NaCl concentrations up to 30% w/v. All isolates grew at ClO_4_^−^ concentrations of 10,000 mg/L, and five isolates also grew at 15,000 mg/L. Phylogenetic analysis identified members of the genera *Psychrobacter, Planococcus, Carnobacterium*, and *Paeniglutamicibacter*, capable of removing the contaminant in percentages between 40% and 52%. In alignment with UN Sustainable Development Goal 3 on promoting health and well-being, this project supports both ecosystem conservation and public health protection. In conclusion, the bacterial strains isolated from Cierva Point marine sediments, Antarctica, represent valuable resources for the bioremediation of ClO_4_^−^ contamination, demonstrating significant potential to reduce and mitigate the pollutant in affected ecosystems.

## Introduction

Antarctica is a remote continent that significantly contributes to the regulation of Earth’s climate, the dynamics of ocean circulation, and carbon sequestration. In particular, its coastal region is characterized by low surface temperatures, extended periods with little to no sunlight, high ultraviolet radiation, aridity, scarce precipitation, extensive sea-ice cover, and limited availability of organic nutrients for most of the year (De Souza et al. 2025; Garzón-Obando 2013). However, the intensification of human presence associated with the expansion of research, tourism, and transportation activities, resulting in the creation and establishment of research stations and transfer systems, may contribute to environmental contamination (Prabagaran et al. 2007; Pereira et al. 2017; Marina-Montes et al. 2020; Q. Xu et al. 2020). ClO_4_^−^ has been detected in trace amounts within Arctic and Antarctic ice layers, indicating its broad occurrence in natural environments (Jiang et al. 2021; Acevedo-Barrios et al. 2025).

ClO_4_^−^ originates from both natural and anthropogenic activities. This contaminant is highly persistent in the environment and, due to its strong solubility and weak adsorption to minerals and organic matter, it exhibits high mobility in surface and groundwater systems (Fang & Naidu 2023; Acevedo-Barrios et al. 2022a; J. Hu et al. 2021), capable of contaminating food and drinking water, ultimately reaching humans through trophic transfer (Jia et al. 2022; Acevedo-Barrios & Olivero-Verbel 2021).

The presence of ClO₄⁻ has been reported in drinking water and multiple food matrices, including vegetables, fruits, dairy products, meats, cereals, beverages, and seafood (J. Hu et al. 2021); as well as in biological samples from adults and infants, such as saliva, urine, maternal blood, breast milk, and in the umbilical cord tissue from newborns (Acevedo-Barrios et al. 2025; Ali et al. 2021; Pleus & Corey 2018). Classified as a potent endocrine disruptor, this compound interferes with iodine uptake by the thyroid gland in higher mammals (Acevedo-Barrios et al. 2025; Cao et al. 2019), affecting their normal development (Acevedo-Barrios et al. 2018, 2025) and increasing the risk of cardiovascular diseases, and negatively influences the reproductive, nervous, and immune systems. Furthermore, ClO_4_^−^ is regarded as a neurotoxic compound capable of inducing teratogenic effects in women during early pregnancy (Acevedo-Barrios et al. 2025; Acevedo-Barrios & Olivero-Verbel 2021; Pleus & Corey 2018).

Due to its ecotoxicological effects, several technologies have been proposed for its reduction in ecosystems. Among them, biological treatments stand out as an efficient, selective, and economically viable alternative, as they replicate natural processes to achieve ClO_4_^−^ reduction (Acevedo-Barrios et al. 2018, 2025). The extreme environmental conditions of the Antarctic ecosystem have favored the evolution of unique adaptive physiological, metabolic, and biochemical mechanisms in native bacteria, enabling them to tolerate and reduce ClO₄⁻ (Acevedo-Barrios et al. 2025).

Antarctica constitutes an ideal ecosystem for the isolation of perchlorate-reducing bacteria (PRB) (Jiang et al. 2021; Acevedo-Barrios et al. 2022b, 2023, 2025). These bacteria, through the enzyme perchlorate reductase (Pcr), reduce ClO_4_^−^ to Chlorate (ClO_3_^−^), and ClO_3_^−^ to Chlorite (ClO_2_^−^). The enzyme chlorite dismutase then transforms ClO_2_^−^ into chloride (Cl^−^) and oxygen (O_2_)(Acevedo-Barrios et al. 2025; Z. Hu et al. 2024).

Cierva Point is located on the Danco Coast, in the northwest Antarctic Peninsula, and it has been designated as ASPA No. 134, with an east-west orientation. Its north-facing slope, extending 1.5 km, has a moderate gradient and rugged terrain primarily covered by grasses, bryophytes, and lichens, and also hosts bird colonies, particularly gentoo penguins (*Pygoscelis papua*) (Casa et al. 2025). The natural occurrence of ClO_4_^−^ has been reported in Cierva Point (Acevedo-Barrios et al. 2022a), increasing the probability of finding PRB in this region. In particular, there has been growing interest in isolating such bacteria from halophilic environments due to their ability to tolerate high concentrations of ClO_4_^−^ and their potential for reducing it (Acevedo-Barrios et al. 2022a, 2022b, 2023, 2025).

Previous studies have demonstrated the ability of Antarctic bacteria from Deception island (Acevedo-Barrios et al. 2022b), Half Moon Island (Acevedo-Barrios et al. 2022b; González-Díaz & Miranda-Peinado 2021), and Horseshoe Island (Acevedo-Barrios et al. 2022b, 2023) to tolerate concentrations of up to 10,000 mg/L of KClO_4_^-^ and to remove it by percentages between 21.6% and 40% (Acevedo-Barrios et al. 2022b) and between 18% and 41% (Acevedo-Barrios et al. 2023).

The bioremediation of ClO_4_^-^ in contaminated ecosystems supports the achievement of SDG 3 by ensuring healthy lives and promoting the well-being of biota. Accordingly, bioremediation strategies, including the use of PRB, contribute to reducing pollution in ecosystems and advancing environmental restoration.

The aim of this study was to isolate and characterize bacterial strains from marine sediments of Cierva Point, Antarctica (ASPA No. 134), and to evaluate their tolerance to NaCl and ClO_4_^−^ as well as their capacity to remove ClO_4_^−^ from the culture medium. To this end, four sequential stages were carried out: (i) isolation and purification of the strains; (ii) morphological, biochemical and molecular characterization; (iii) NaCl and ClO_4_^−^ susceptibility assays; and (iv) ClO_4_^−^ removal assays. In this context, the removal of ClO_4_^−^ using bacterial strains isolated from marine sediments in Cierva Point, Antarctica (ASPA No. 134), with biotechnological potential for removing this contaminant, represents the establishment of the first Antarctic perchlorate-removing bacterial bank. This pioneering bank opens new opportunities for the development of bioremediation technologies, expands knowledge of microbial diversity in ecosystems, and provides a foundation for future research aimed at innovative solutions to emerging contaminants, while contributing to the fulfillment of SDG 3 by promoting health and well-being in ecosystems.

## Materials and methods

### Study area and sample collection

**Fig. 1:**
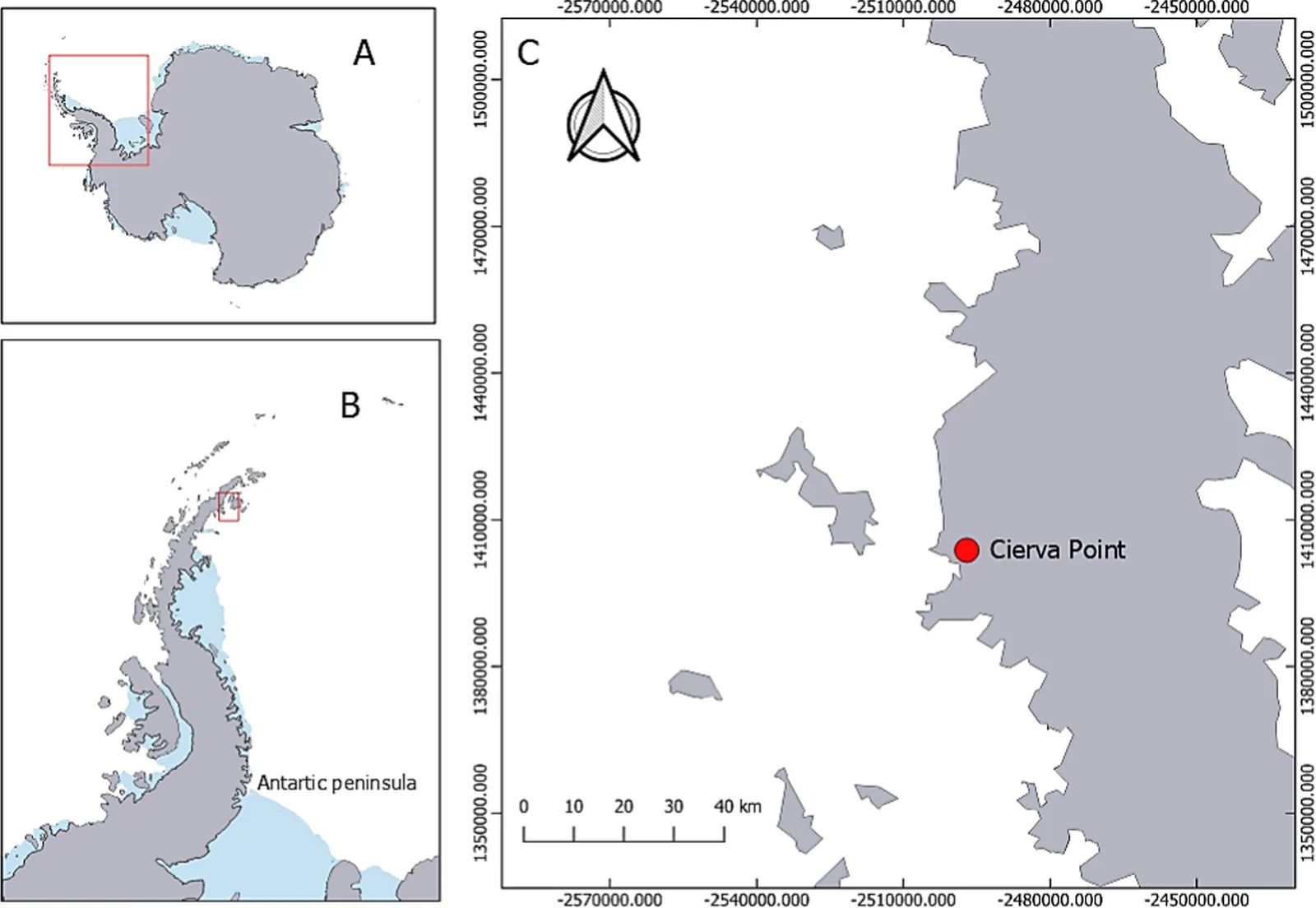
Map of the area under study showing the sediment sampling site in Cierva Point, Antarctica. **A** Antarctic continent. **B** Antarctic Peninsula. **C** Cierva point

Marine sediment samples were collected during the 6th Colombian Scientific Expedition to Antarctica, Austral Summer (2019–2020) in February 2020 within the Cierva Point ASPA (64°09′00"S, 60°53′00"W; No. 134) (Fig. 1). Around 50 g of each sample were collected in triplicate using sterile dredges and stored in 15 mL Falcon tubes, following the protocol described by Acevedo-Barrios et al. (2022b, 2023). Sampling was carried out under the environmental requirements of the Protocol on Environmental Protection to the Antarctic Treaty and the Management Plan for ASPA No. 134, within the framework of the Colombian Antarctic Program coordinated by the Colombian Ocean Commission (CCO) in cooperation with the Spanish Polar Committee. The corresponding permit documentation is held by the Colombian Antarctic Program and can be provided to the Editor upon request.

### Isolation and planting conditions

Isolation, purification, and preservation were carried out as described by Acevedo-Barrios et al. (2018, 2022b, 2023, 2024). Colonies with distinguishable macroscopic morphological traits were selected and subjected to re-streaking procedures on LB agar medium modified with seawater.

### Microbial characterization

#### Morphological and biochemical characterization

Morphological characterization of the strains included macroscopic identification, performed through direct observation of the colonies, and microscopic identification using Gram staining with a Nikon E100 optical microscope, following the instructions of Acevedo-Barrios et al. (2022b, 2023, 2024).

For biochemical identification, BBL™ Crystal™ Kit ID tests were carried out according to the manufacturer’s instructions. Additionally, catalase and oxidase tests were performed following the original methodological descriptions of Reiner (2010) and Kovács (1956), respectively, under the conditions previously applied by Acevedo-Barrios et al. (2018, 2022b, 2023, 2024).

#### 16S rRNA gene sequencing and phylogenetic analysis

Bacterial isolates were identified via near-full-length 16S rRNA gene sequencing. Genomic DNA was extracted and purified prior to amplification. Polymerase chain reaction (PCR) amplification targeted an approximately 1,500 bp region of the 16S rRNA gene using universal bacterial primers 27 F (5’-AGAGTTTGATCCTGGCTCAG-3) and 1492R (5’-GGTTACCTTGTTACGACTT-3) (Weisburg et al. 1991). Amplicon quality was verified by agarose gel electrophoresis, and PCR products were subsequently purified and subjected to bidirectional Sanger sequencing at CorpoGen (Bogotá, Colombia). To ensure high base-calling accuracy and comprehensive coverage across the entire fragment, sequencing reactions employed both external PCR primers (27F and 1492R) and internal primers: 337 F (5’-GACTCCTACGGGAGGCWGCAG-3), 518 F 5’-CCAGCAGCCGCGGTAATACG-3), 800R (5’-TACCAGGGTATCTAATCC-3), and 1100R 5’-GGGTTGCGCTCGTTG-3). Consensus sequences, with a mean assembled length of approximately 1,400 bp, were reconstructed by trimming and assembling overlapping forward and reverse reads using Geneious software (Biomatters, New Zealand). Taxonomic assignment was performed via BLASTn against the NCBI RefSeq RNA database, assessing percentage identity, query coverage, alignment length, and closest reference matches. Species-level identification required (>98.65%) sequence identity to validly published type strains within genera possessing reliable 16S rRNA resolution; otherwise, isolates were conservatively designated at the genus level. Complementary phylogenetic analyses were conducted using the leBIBI IV SSU-rDNA tool (Université de Lyon), which integrates the BIBI DB mk45 database (December 2022 release) with Kalign v3.3, FastTree v2.1.11, CIAlign 1.0.9, Fastroot v1.5, and ToyTree v1. Finally, multiple sequence alignments of each consensus sequence with the thirty most similar reference strains were generated using the MUSCLE algorithm.

The GenBank accession numbers for the sequences of the isolates are: UTB_250 PQ184777, UTB_251 PQ184778, UTB_252 PQ184779, UTB_253 PQ184780, UTB_254 PQ184781, UTB_256 PQ184782, UTB_140 PX461653, UTB_285 PX461654, UTB_270 PX461655, UTB_271 PX461656, UTB_143 PX461657.

### Sodium chloride (NaCl) susceptibility test

Sodium Chloride tolerance was determined by incubating the isolates in LB medium supplemented with increasing concentrations of NaCl (3.5%, 15%, 20%, and 30% w/v), in triplicate. Under each condition, cultures were incubated at 10 °C for 7 d, and turbidity was visually assessed as an indicator of growth. A strain was classified as tolerant (R) at a given concentration only when visible turbidity was recorded in all three replicate tubes, and as susceptible (S) when no turbidity was observed in any of them; no discordant replicates were obtained under any of the conditions tested. The same criterion was applied in the perchlorate susceptibility assay. This procedure follows that recommended by Acevedo-Barrios et al. (2018, 2022b, 2023, 2024).

### Perchlorate susceptibility test

The ability of the isolates to tolerate increasing concentrations of potassium perchlorate (KClO_4_^−^) 700, 5,000, 10,000, and 15,000 mg/L dissolved in LB medium solutions prepared with sterile distilled water, were evaluated under similar conditions to the NaCl susceptibility test. Bacteria were inoculated in triplicate into sterile tubes containing 5 mL of medium, with 20 μL of a pre-culture adjusted to an optical density at 600 nm (OD600) of 0.6, corresponding to an initial OD600 of approximately 0.0024 in the assay system after the 1:250 dilution. Growth was assessed exclusively by visual inspection of turbidity at the end of incubation; no intermediate or quantitative OD measurements were recorded. After 7 d at 10 °C, the presence of visible turbidity in all three replicate tubes was recorded as evidence of KClO_4_^−^ tolerance, in accordance with the procedure proposed by Acevedo-Barrios et al. (2018, 2022b, 2023, 2024).

### Evaluation of perchlorate removal by isolates

The ClO_4_^−^ removal capacity was evaluated in LB medium supplemented with 10,000 mg/L of KClO_4_^-^. Cultures were incubated for 7 d at 10 °C, after which the remaining ClO_4_^−^ concentration was quantified using a ClO_4_^−^ ion-selective electrode (Orion-93, Thermo Scientific), calibrated with matrix-matched standards prepared in sterile, uninoculated LB broth rather than in water, over a linear range of 10 to 15,000 mg/L ClO_4_^−^. A commercial ionic strength adjustment solution (Thermo Scientific Orion Perchlorate ISA) was added to all standards and samples at 2% v/v prior to measurement to maintain a uniform ionic background. An uninoculated abiotic control consisting of LB medium supplemented with 10,000 mg/L KClO_4_^−^ was incubated in parallel under identical conditions (7 d, 10 °C, pH 7.0±0.5) and measured with the same electrode at the beginning and at the end of the assay, in order to distinguish biologically mediated removal from abiotic loss, adsorption or electrode drift. Removal was calculated as the percentage decrease between the initial and final ClO_4_^−^ concentrations, and is reported as overall removal at the system level rather than as cell-specific activity, since biomass normalization was not performed.The removal assay was performed in triplicate for each isolate, with each tube considered an independent experimental unit. Removal percentages were calculated independently for each replicate tube from the nominal initial concentration (10,000 mg/L) and the final ClO_4_^-^ concentration measured in that tube, and are reported as mean ± standard deviation (SD) of the three biological replicates. Differences among isolates were evaluated inferentially as described in Section 2.7. Because the electrode measures the decrease in ClO_4_^−^ concentration in the culture medium but does not identify its transformation products, the results are reported throughout as perchlorate removal and not as perchlorate reduction, in accordance with the procedure proposed by Acevedo-Barrios et al. (2018, 2022b, 2023, 2024).

### Statistical analysis

Perchlorate removal assays were performed in triplicate, using three independently inoculated tubes per strain, which were treated as biological replicates. Removal percentages were calculated for each replicate and are expressed as mean ± standard deviation (SD). Normality of residuals was assessed with the Shapiro–Wilk test (Shapiro & Wilk 1965) and homogeneity of variances with the median-centred Levene test (Levene 1960). Differences in removal percentage among isolates were evaluated by one-way analysis of variance (ANOVA), followed by Tukey’s honestly significant difference (HSD) test for multiple comparisons (Tukey 1949). A parametric approach was selected because the design involves a single fixed factor (bacterial strain) with independent replicates and a continuous response variable, the conditions under which one-way ANOVA is the appropriate test (Zar 2010). Tukey’s HSD was preferred over multiple pairwise t-tests because it controls the family-wise error rate across all 55 comparisons while retaining greater power than the Bonferroni correction in a balanced design (Tukey 1949; Zar 2010). Because the number of replicates per group was small, the distribution-free Kruskal–Wallis test (Kruskal & Wallis 1952) was applied in parallel, and the agreement between both approaches was taken as evidence of the robustness of the conclusions. Homogeneous groups were defined from the Tukey HSD output and are indicated by letters in Fig. 3. Effect size is reported as eta squared (η^2^). A significance level of α = 0.05 was adopted throughout. All analyses were performed in Python 3.11 using the SciPy library v1.17 (Virtanen et al. 2020).

## Results

### Morphological and biochemical characterization

The results of the biochemical and morphological characterization of the strains isolated from Cierva Point are presented in Tables 1 and 2. The predominant morphological feature corresponded to coccobacilli. Likewise, most of the isolated strains were Gram-negative (−), belonging to the genus *Psychrobacter*; The Gram-positive (+) isolates belonged to the genera *Planococcus*, *Carnobacterium*, and *Paeniglutamicibacter*. Biochemical characterization revealed the presence of the enzyme catalase in all samples and cytochrome oxidase C in certain bacterial strains.

**Table 1:** Morphological and biochemical characteristics of Gram-positive (+) isolated strains

Characteristic	UTB-143	UTB-250	UTB-251	UTB-252	UTB-253
Closest relative (% 16S rRNA sequence similarity)	*Planococcus* *halocryophilus* (99)	*Carnobacterium* *iners* (99)	*Carnobacterium* *Iners* (98)	*Carnobacterium* *iners* (98)	*Paeniglutamicibacter* *Antarcticus* (99)
Source	Cierva point	Cierva point	Cierva point	Cierva point	Cierva point
Color of colony	Beige	Cream	Cream	Cream	Yellow
Morphology	Cocobacilli	Cocobacilli	Cocobacilli	Cocobacilli	Bacillococci
Halotolerant	+	+	+	+	+
Psychrophilic	+	+	+	+	+
Motility	−	−	−	−	−
Gram staining	+	+	+	+	+
Endospore	−	−	−	−	−
Spore position	−	−	−	−	−
Oxidase	−	+	+	+	+
Catalase	+	+	+	+	+
Arginine	−	+	−	−	−
Fluorescent negative control	+	−	−	−	−
Sucrose	−	−	−	−	−
Mannitol	−	−	−	−	−
p-n-p-phosphate	−	−	−	−	+
p-n-p -ß-D-glucoside	−	−	−	−	+
p-n-p- ß-D-cellobioside	−	−	−	−	−
Esculin	−	+	−	−	+
Urea	−	−	−	+	+
Glycine	−	−	−	−	−
Lactose	−	−	−	−	−
ONPG & p-n-p-a-D	−	−	−	−	+
4 MU- ß-D-glucoside	−	−	−	−	−
L-valine	−	−	−	−	−
L-phenylalanine-AMC	−	−	−	−	−
4MU-a-D-glucoside	−	−	−	−	−
L-pyroglutamic acid -AMC	−	−	−	−	−
L-tryptophan-AMC	−	−	−	−	−
L-arginine	−	−	−	−	−
4MU-N-acetyl- ß-D-glucosaminide	−	−	−	−	−
4MU-phosphate	−	−	−	−	−
4 MU- ß-D-Glucuronide	−	−	−	−	−
L-isoleucine	−	−	−	−	−
Trehalose	−	−	−	−	−
Methyl-a & ß-glucoside	−	−	−	−	−
Maltotriose	−	−	−	−	−
Glycerol	−	−	−	−	−
Fructose	−	−	−	−	−
Proline & leucine–p- nitroanilide	−	−	+	−	+
p-n-p-a-D maltoside	−	−	−	−	+
Arabinose	+	−	−	−	−

**Table 2:** Morphological and biochemical characteristics of Gram-negative (−) isolated strains

Characteristic	UTB-254	UTB-256	UTB-140	UTB-270	UTB-271	UTB-285
Closest relative (% 16S rRNA sequence similarity)	*Psychrobacter arcticus* (99)	*Psychrobacter arcticus* (98)	*Psychrobacter glacincola* (99)	*Psychrobacter maritimus* (99)	*Psychrobacter maritimus* (99)	*Psychrobacte* *glacincola* (98)
Source	Cierva point	Cierva point	Cierva point	Cierva point	Cierva point	Cierva point
Color of colony	Cream	Cream	Beige	Beige	Beige	Beige
Morphology	Cocobacilli	Cocobacilli	Cocobacilli	Cocobacilli	Cocobacilli	Cocobacilli
Halotolerant	+	+	+	+	+	+
Psychrophilic	+	+	+	+	+	+
Motility	−	−	−	−	−	−
Gram staining	−	−	−	−	−	−
Endospore	−	−	−	−	−	−
Spore position	−	−	−	−	−	−
Oxidase	+	+	+	+	+	+
Catalase	+	+	+	+	+	+
Arginine	−	−	−	−	−	−
Arabinose	−	−	−	−	−	−
Sucrose	−	−	−	−	−	−
Mannitol	−	−	−	−	−	−
p-n-p-phosphate	−	−	+	−	−	−
p-n-p α-ß-glucoside	−	−	−	−	−	−
p-n-p- ß-galactoside	−	−	−	−	−	−
Esculin	−	−	+	+	+	+
Urea	−	−	−	−	−	−
Glycine	−	−	−	−	−	−
Lysine	−	−	−	−	−	−
Mannose	−	−	−	+	−	−
Melibiose	−	−	−	−	−	−
Rhamnose	−	−	−	−	−	−
Sorbitol	−	−	−	−	−	−
Adonitol	−	−	+	−	+	−
Galactose	−	−	−	−	−	−
Inositol	−	−	−	−	−	−
Proline nitroanilide	−	−	−	−	−	−
p-n-p bis -phosphate	−	−	−	−	−	−
p-n-p-xyloside	−	−	−	−	−	−
p-n-p-α-arabinoside	−	−	−	−	−	−
p-n-p-phosphorylcholine	−	−	−	−	−	−
p-n-p- ß-glucuronide	−	−	−	−	−	−
p-n-p-N-acetyl glucosaminide	−	−	−	−	−	−
γ-L-glutamyl p-nitroanilide	−	−	−	−	−	−
p-nitro-DL-phenylalanine	−	−	−	+	−	−
Citrate	−	−	−	−	−	−
Malonic acid	−	−	−	−	−	−
Tripheynyl tetrazolium chloride	−	−	−	−	−	−

### Molecular characterization of isolated strains

**Fig. 2:**
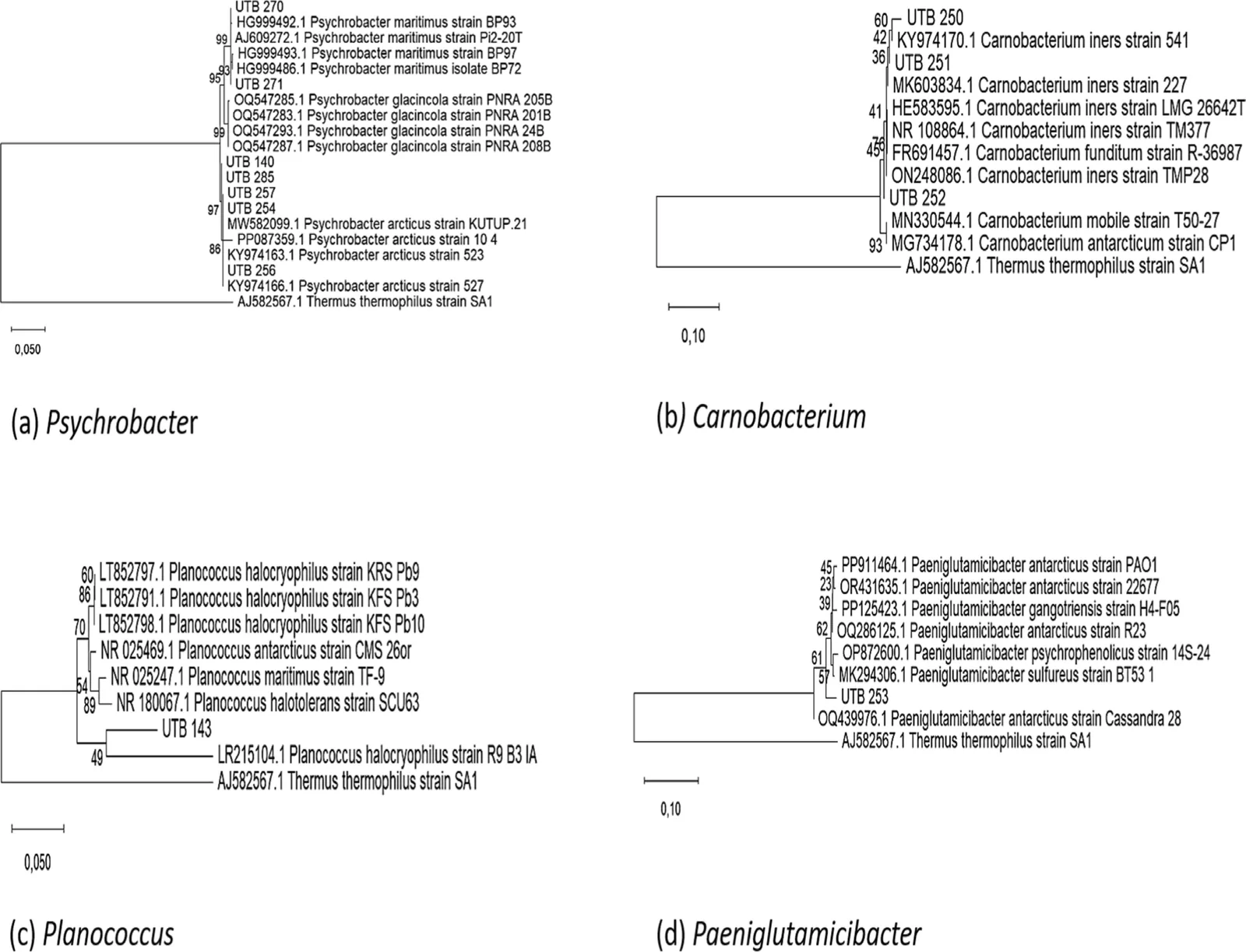
Phylogenetic reconstruction of the 16S rRNA gene using the Neighbor-Joining method with the Tamura-Nei (TN93) distance model and 1,000 bootstrap replicates, used to determine the taxonomic identity of the *Psychrobacter, Carnobacterium, Planococcus* and *Paeniglutamicibacter* Genus Isolated in Cierva Point, Antarctica. **a** *Psychrobacter* **b** *Carnobacterium* **c** *Planococcus ****d**** Paeniglutamicibacter*

Molecular identification of the isolated strains showed that the genus *Psychrobacter* predominates in the sampling area, accounting for approximately 54% of the isolates, 27% of the strains correspond to *Carnobacterium* sp. closely related to *C. iners*, while the remaining percentage is equally divided between the genera *Planococcus* and *Paeniglutamicibacter*. This distribution is illustrated in the phylogenetic tree of the molecularly characterized species (see Fig. 2).

### Susceptibility test to NaCl and potassium perchlorate

Sodium chloride susceptibility assays showed that most of the evaluated strains were able to grow at concentrations of up to 15% w/v NaCl, while strains UTB-140, UTB-143, UTB-285, UTB-270, and UTB-271 tolerated up to 30% w/v NaCl (see Table 3). Regarding ClO₄⁻ susceptibility, all evaluated strains grew at 700, 5,000 and 10,000 mg/L, whereas growth at 15,000 mg/L was restricted to strains UTB-140, UTB-143, UTB-285, UTB-270 and UTB-271 (see Table 3), indicating that they tolerate ClO₄⁻ concentrations well above the mean value recorded in marine sediments from Cierva Point (131.667 ± 7.635 mg/kg; n = 240), although both figures are not directly comparable, since the environmental value refers to sediment and the assay concentrations to liquid medium (Acevedo-Barrios et al. 2022a).

**Table 3:** Percentage of susceptibility test to NaCl and potassium perchlorate concentrations from marine sediment samples taken from Cierva Point

Conditions/ concentrations	NaCl (%w/v)				KClO_4_^−^(mg/L)			
	3,5	15	20	30	700	5000	10000	15000
UTB-140	R	R	R	R	R	R	R	R
UTB-143	R	R	R	R	R	R	R	R
UTB-250	R	R	S	S	R	R	R	R
UTB-251	R	R	S	S	R	R	R	R
UTB-252	R	R	S	S	R	R	R	R
UTB-253	R	R	S	S	R	R	R	R
UTB-254	R	R	S	S	R	R	R	R
UTB-256	R	R	S	S	R	R	R	R
UTB-270	R	R	R	R	R	R	R	R
UTB-271	R	R	R	R	R	R	R	R
UTB-285	R	R	R	R	R	R	R	R

*S* susceptible, *R* resistance.

After 7 d of contact at 10 °C, inoculated with a pre-culture adjusted to OD600 = 0.6, at pH 7.0±0.5. R, resistant (growth observed); S, susceptible (no growth observed). Assays were performed in triplicate

Table 3. Percentage of Susceptibility Test to NaCl and Potassium Perchlorate concentrations from marine sediment samples taken from Cierva Point. After 7 d of contact at 10 °C, inoculated with a pre-culture adjusted to OD600 = 0.6, at pH 7.0±0.5. R, resistant (growth observed); S, susceptible (no growth observed). Assays were performed in triplicate.

### Evaluation of perchlorate removal by isolates

**Fig. 3:**
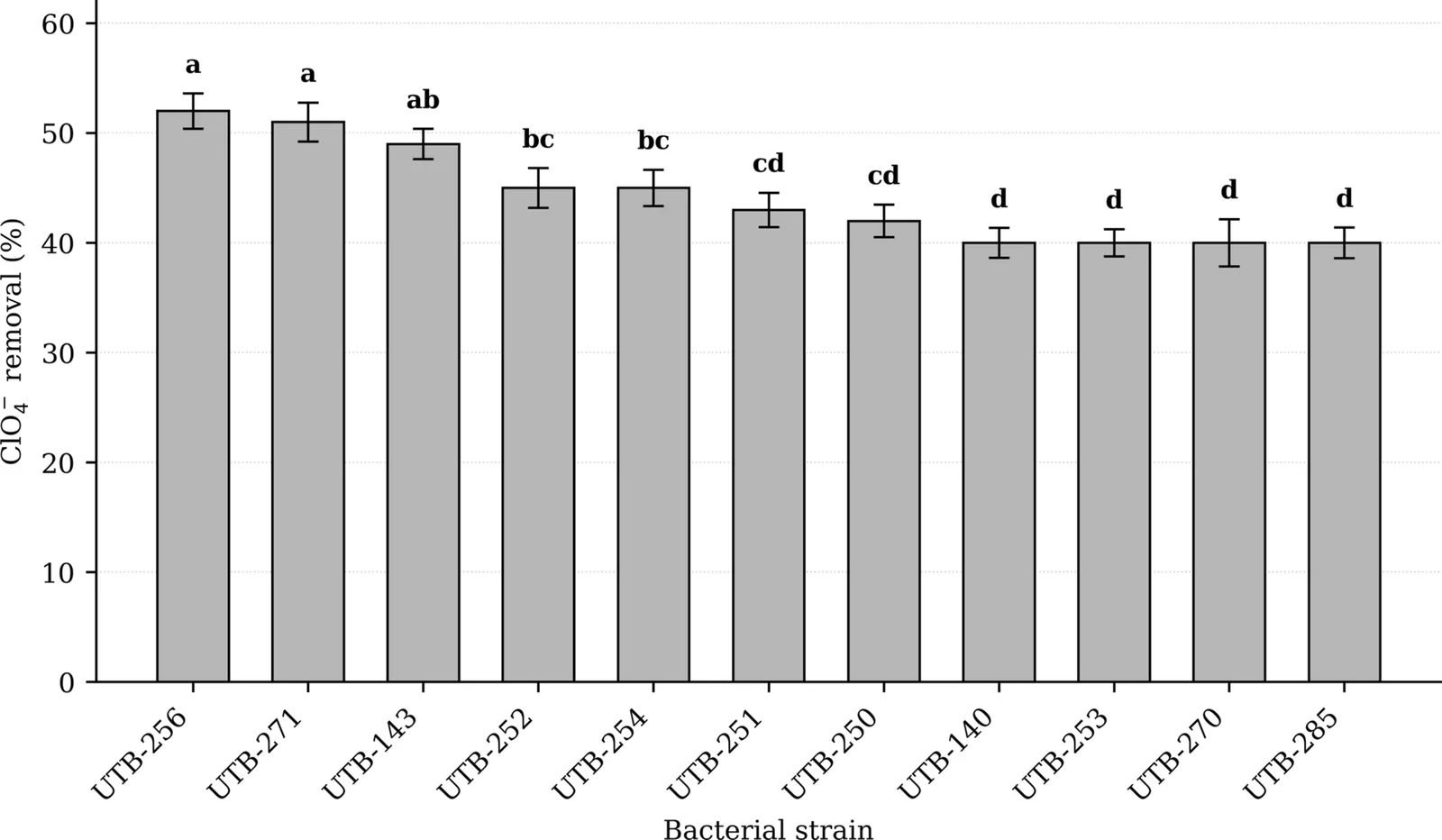
Percentage removal of KClO_4_^-^ concentration from marine sediment samples taken from Cierva Point. Effect after 7 d of contact at 10 °C, inoculated with a pre-culture adjusted to OD600 = 0.6, at pH 7.0±0.5. Bars represent the mean of three independently inoculated replicates (n = 3) and error bars represent the standard deviation. Different letters above the bars indicate statistically significant differences among strains (one-way ANOVA followed by Tukey’s HSD test, p < 0.05)

The bacterial strains were exposed for 7 d to different concentrations of potassium perchlorate (KClO_4_^−^). They demonstrated the ability to remove KClO_4_^-^ at concentrations of 10,000 mg/L, with removal percentages ranging from 40.0 ± 1.3% to 52.0 ± 1.6% (mean ± SD, n = 3). Removal percentages differed significantly among the isolates (one-way ANOVA, F(10,22) = 24.28, p < 0.001, η^2^ = 0.92), a result confirmed by the Kruskal–Wallis test (H = 28.11, p = 0.002). Variances were homogeneous across groups (Levene, p = 0.999), whereas the Shapiro–Wilk test on residuals indicated a marginal departure from normality (p = 0.025); the agreement between the parametric and non-parametric tests supports the robustness of the comparison. Tukey’s HSD test identified strains UTB-256 (52.0 ± 1.6%), UTB-271 (51.0 ± 1.8%) and UTB-143 (49.0 ± 1.4%) as the most efficient isolates, with removal percentages significantly higher than those of UTB-140, UTB-253, UTB-270 and UTB-285 (40.0 ± 1.3% to 40.0 ± 2.2%; p < 0.001 in all cases), whereas the difference between UTB-256 and UTB-143 was not statistically significant (p = 0.14). The mean removal percentages, their dispersion and the homogeneous groups defined by the Tukey HSD test are summarized in Table 4; the individual replicate values and the complete matrix of pairwise comparisons are provided as Supplementary Material. No decrease in ClO_4_^−^ concentration was observed in the uninoculated abiotic control. Strain UTB-256 (*Psychrobacter arcticus*) showed the highest mean removal percentage at 52%, while strains UTB-143 (*Planococcus halocryophilus*) and UTB-271 (*Psychrobacter maritimus*) achieved removal percentages of 49% and 51%, respectively (see Fig. 3).

**Table 4:** Perchlorate removal by the isolated strains after 7 d of contact at 10 °C in LB medium supplemented with 10,000 mg/L KClO_4_^−^

Strain	Molecular identification	Removal (%, mean ± SD)	SEM	Group
UTB-256	*Psychrobacter arcticus*	52.0 ± 1.6	0.93	a
UTB-271	*Psychrobacter maritimus*	51.0 ± 1.8	1.02	a
UTB-143	*Planococcus halocryophilus*	49.0 ± 1.4	0.80	ab
UTB-252	*Carnobacterium iners*	45.0 ± 1.8	1.04	bc
UTB-254	*Psychrobacter arcticus*	45.0 ± 1.7	0.95	bc
UTB-251	*Carnobacterium iners*	43.0 ± 1.6	0.91	cd
UTB-250	*Carnobacterium iners*	42.0 ± 1.5	0.85	cd
UTB-140	*Psychrobacter glacincola*	40.0 ± 1.4	0.78	d
UTB-253	*Paeniglutamicibacter antarcticus*	40.0 ± 1.3	0.72	d
UTB-270	*Psychrobacter maritimus*	40.0 ± 2.2	1.25	d
UTB-285	*Psychrobacter glacincola*	40.0 ± 1.4	0.80	d

Values are the mean ± standard deviation of three independently inoculated replicates (n = 3) SEM, standard error of the mean. Strains sharing a letter in the Group column do not differ significantly (one-way ANOVA, F(10,22) = 24.28, p < 0.001, followed by Tukey’s HSD test, α = 0.05)

The distribution of these removal percentages and the homogeneous groups resulting from the post-hoc comparison are shown graphically in Fig. 3.

## Discussion

### Microscopic, biochemical, and molecular characterization

Taxonomic characterization through analysis of the 16S rRNA gene sequence and phylogenetic reconstruction indicated that the eleven isolates belong to the genera *Psychrobacter*, *Carnobacterium*, *Planococcus*, and *Paeniglutamicibacter*. The predominance of the genus *Psychrobacter* is consistent with previous reports (González-Díaz & Miranda-Peinado 2021; Acevedo-Barrios et al. 2022b, 2023), these psychrophilic bacteria tested positive for both catalase and oxidase, as this genus is dominant and found across a wide variety of moist, saline, and cold habitats, along with warm and moderately saline environments (Acevedo-Barrios et al. 2022b). Species such as *Psychrobacter arcticus*, *Psychrobacter glacincola*, and *Psychrobacter maritimus* have also been identified in Arctic glaciers (Zeng et al. 2016).

Meanwhile, the genus *Carnobacterium* has been identified in sediments of Antarctic lakes, Arctic permafrost, and Antarctic coastal sandy soils; These bacteria are Gram-positive, non-motile facultative anaerobes, non-spore-forming, exhibiting cellular arrangements as single rods, paired cells, or small chains (Kosiorek et al. 2024). Three strains belonging to this genus were identified, all belonging to the species *Carnobacterium iners*, with positive catalase and oxidase. The genus *Planococcus* is characterized as Gram-positive and halotolerant, belonging to the phylum *Bacillota*. Commonly detected across various Antarctic habitats, it is particularly abundant in cold habitats such as Arctic permafrost, comprising about 5.8% of the bacterial community (Ay et al. 2025). The characterized strain belongs to the species *Planococcus halocryophilus*, a species previously identified on Horseshoe Island (Margaret Bay), Antarctica (Ay et al. 2025).

The strain *Paeniglutamicibacter antarcticus* was successfully isolated. This genus of Gram-positive bacteria is characterized by containing peptidoglycan type A4α with L-lysine as the dibasic amino acid (Lys–Glu; A11.54), and by having MK-9 or MK-10 as the predominant menaquinones. This species has been reported in parts of King George Island, Antarctica (Sakdapetsiri et al. 2021). These extremophilic bacteria’s principal application involves biotransformation processes that enable both *in situ* and *ex situ* bioremediation of ecosystems, allowing the treatment of unsaturated zones and contaminated soils (Acevedo-Barrios et al. 2025).

### Evaluation of perchlorate removal

The species *Psychrobacter arcticus* (UTB 256) and *Psychrobacter maritimus* (UTB 271) achieved removal of more than 50% of KClO₄⁻ in the evaluated ecosystem. In turn, *Psychrobacter glacincola* (UTB140 - UTB 285), *Psychrobacter maritimus* (UTB 270) and *Paeniglutamicibacter antarcticus* (UTB 253) reached a removal percentage of 40%. The remaining isolates exhibited intermediate removal percentages.

It should be noted that perchlorate tolerance and perchlorate removal are biologically distinct traits: the susceptibility assay was used to define the working concentration for the removal assay, and tolerance alone should not be interpreted as evidence of biodegradation. Tolerance was recorded here as a binary resistant/susceptible criterion based on visual turbidity, which limits the discrimination among isolates classified within the same category. Future work should incorporate quantitative OD600 measurements and concentration-response growth profiles, which would allow a more objective comparison of perchlorate tolerance and a better-supported prioritization of strains with biotechnological interest. Five of the isolates tolerated ClO₄⁻ concentrations of up to 15,000 mg/L, and removal percentages ranged from 40% to 52% at 10,000 mg/L. In particular, strain UTB-256 exhibited 52% removal after one week of exposure to 10,000 mg/L and incubation at 10 °C, positioning it as a promising candidate for bioremediation applications. Compared with previously reported percentages for perchlorate-reducing bacteria (PRBs) in Antarctic environments, which range between 21.5% and 22% (González-Díaz & Miranda-Peinado 2021), 21.6% and 40% (Acevedo-Barrios et al. 2022b), and 18% and 41% (Acevedo-Barrios et al. 2023), these bacterial strains showed higher mean removal percentages. However, because the analytical procedure has not yet been validated in an equivalent experimental matrix across studies, this comparison should be regarded as preliminary. On the other hand, extremophilic bacteria isolated from other ecosystems, such as hypersaline soils of the Colombian Caribbean, showed comparable reduction profiles. For example, bacteria of the genus *Bacillus* were able to reduce between 10% and 25% of ClO₄⁻ (Acevedo-Barrios et al. 2018, 2024).

### Project contribution to the SDG

The main route of human exposure to perchlorate (ClO₄⁻) is through the ingestion of contaminated food and drinking water. In certain contexts, this compound can also enter the body via the respiratory tract through the inhalation of contaminated dust (Acevedo-Barrios et al. 2025; Pleus & Corey 2018). The frequent presence of ClO₄⁻ in commonly consumed foods such as fruits, rice, milk, and dairy products is documented in several studies, representing a direct threat to public health (Jia et al. 2022; Chen et al. 2021; Niziński et al. 2021). ClO₄⁻ is a contaminant with well-recognized adverse effects, including toxicity and potential carcinogenicity, with a particular impact on thyroid cells (Ali et al. 2021). Its most documented mechanism of action is the competitive inhibition of the sodium-iodide symporter (NIS), which reduces iodide uptake by the thyroid gland, leading to hormonal dysfunctions that may affect metabolism, neurological development, and growth, especially in children and pregnant women (Acevedo-Barrios et al. 2025; Acevedo-Barrios & Olivero-Verbel 2021).

In this context, this article gains relevance as it is directly aligned with Sustainable Development Goal 3 (Good Health and Well-being), one of the pillars of the United Nations 2030 Agenda. This goal seeks to ensure healthy lives and promote well-being for all people at all stages of life (United Nations, n.d.). The proposed project contributes to this objective through a preventive and mitigation-oriented approach, by developing biotechnological solutions aimed at reducing or eliminating the presence of toxic compounds such as ClO₄⁻ in the environment.

**Fig. 4:**
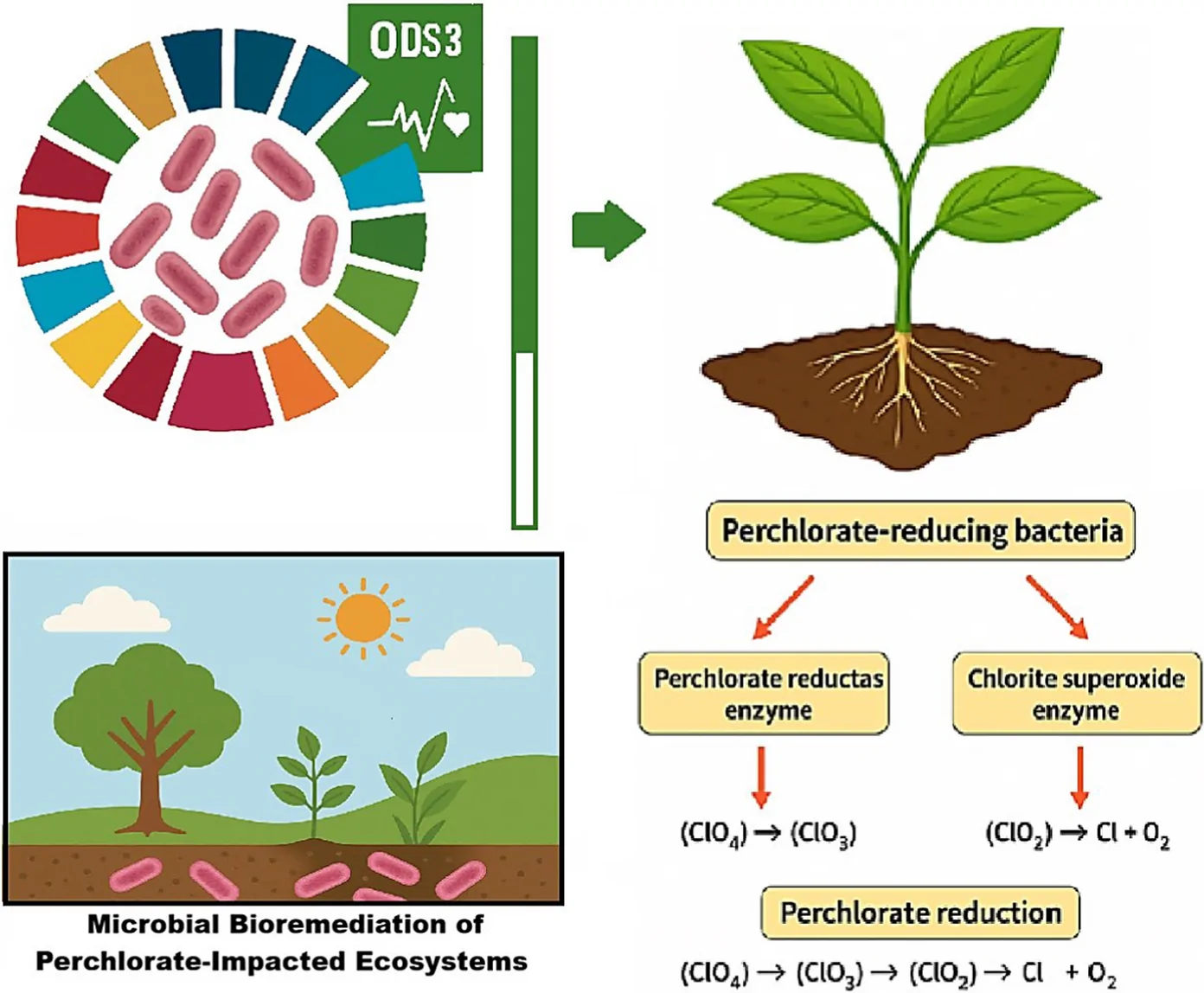
Contribution of the project to SDG 3. Microbial metabolism of ClO₄⁻ reduction applied in the bioremediation of contaminated ecosystems

In particular, the use of perchlorate-degrading bacteria represents an innovative and sustainable bioremediation strategy, enabling the effective degradation of this contaminant without the generation of hazardous secondary residues (Acevedo-Barrios & Olivero-Verbel 2021). The bacteria reduce ClO₄⁻ by employing it as a terminal electron acceptor while oxidizing a wide range of substrates. This process depends on two key enzymes: perchlorate reductase and chlorite dismutase. The metabolic reduction pathway (see Fig 4) involves the sequential conversion of perchlorate to chlorate (ClO₃⁻), then to chlorite (ClO₂⁻), and finally to chloride (Cl⁻) and molecular oxygen (O₂). It should be emphasized that neither these enzymatic activities nor their encoding genes were assessed in the present study; the pathway is presented as the biological context motivating the search for such isolates (Xu et al. 2003; Anupama et al. 2015; Matsubara et al. 2017; Acevedo-Barrios et al. 2025)

This methodology stands out for its low cost, its capacity for in situ treatment, and its potential to naturally restore contaminated ecosystems (Copetti et al. 2022). By removing a highly toxic chemical contaminant from water and soil, it protects human health, reduces chronic exposure to hazardous compounds, and improves the quality of life in affected communities. Therefore, this project not only addresses an environmental issue but also responds to an urgent need for the environmental health of ecosystems, contributing to the fulfillment of SDG 3 from a multidisciplinary perspective that integrates science, technology, and social well-being. Furthermore, it promotes a sustainable development model based on prevention, the responsible management of natural resources, and the protection of collective health.

## Conclusion

The native Antarctic bacteria characterized in this study removed perchlorate (ClO₄⁻) from the culture medium at percentages higher than those reported in previous studies conducted under comparable environmental conditions. The strains isolated from marine sediments in the Antarctic Specially Protected Area (ASPA) Cierva Point showed removal percentages ranging from 40 to 52% at 10,000 mg/L, and tolerated NaCl concentrations of up to 30% w/v; five of the isolates also grew at 15,000 mg/L KClO₄⁻. This work was conceived as a screening study; optimization of the removal process (carbon source, electron donor, C/N ratio, temperature and contact time) was not addressed and remains a priority for future work.

From a taxonomic perspective, a microbial diversity composed of psychrophilic and halotolerant genera was identified. The predominant genus was *Psychrobacter*, with species such as *P. arcticus*, *P. maritimus*, and *P. glacincola*, followed by *Carnobacterium*, represented by three strains of *C. iners.* Additionally, strains of *Paeniglutamicibacter antarcticus* and *Planococcus halocryophilus* were characterized, thereby expanding current knowledge of Antarctic microbiota with bioremediation potential.

These findings highlight the biotechnological value of Antarctic bacteria in mitigating emerging contaminants, particularly in fragile ecosystems such as polar regions. Their application in bioremediation processes represents a promising tool for the removal of toxic compounds such as perchlorate, directly contributing to the achievement of Sustainable Development Goal (SDG) 3: Good Health and Well-Being, by reducing risks associated with environmental exposure to this contaminant for both biota and human health (U.S. Environmental Protection Agency 2025; World Health Organization 2016).

This research provides relevant evidence on the capacity of Antarctic bacterial strains to be used in bioremediation strategies adapted to extreme conditions, laying the groundwork for future studies aimed at optimizing their large-scale application. In addition to expanding knowledge on Antarctic microbial diversity and its bioremediation potential, this study directly contributes to the achievement of SDG 3 by offering a biotechnological solution to mitigate the risks associated with ClO₄⁻ exposure, an emerging contaminant with well-documented adverse effects on human health. The ability of these native strains to remove ClO₄⁻ under extreme conditions represents not only a scientific advancement but also a practical tool for protecting public health, particularly in contexts where conventional technological resources are limited or less effective. Thus, the development of strategies based on Antarctic microorganisms has both environmental and health impacts, reinforcing the need for integrated approaches that bridge science, health, and sustainability.

## Data Availability

Original data can be provided upon reasonable request

## Abbreviations

ASPA: Antarctic specially protected area
(ClO_4_^−^): Perchlorate
LB: Luria-bertani medium
NaCl: Sodium chloride
NCBI: National center for biotechnology information
PCR: Polymerase chain reaction
BLAST: Basic local alignment search tool
PRB: Perchlorate reducing bacteria
SDG: Sustainable development goal
d: Days
